# Randomized controlled trial of a teleconference fatigue management plus physical activity intervention in adults with multiple sclerosis: rationale and research protocol

**DOI:** 10.1186/1471-2377-12-122

**Published:** 2012-10-16

**Authors:** Matthew Plow, Marcia Finlayson, Robert W Motl, Francois Bethoux

**Affiliations:** 1 Department of Biomedical Engineering, Department of Physical Medicine and Rehabilitation, Cleveland Clinic Lerner Research Institute, 9500 Euclid Ave, ND-20, Cleveland, OH, 44195, USA; 2 School of Rehabilitation Therapy, Queen’s University, Louise D. Acton Building, 31 George Street, Kingston, Ontario, K7L 3N6, Canada; 3 Department of Kinesiology and Community Health, University of Illinois Urbana-Champaign, 350 Freer Hall, 906 South Goodwin Avenue, Urbana, IL, 61801, USA; 4 Mellen Center for Multiple Sclerosis Treatment and Research, Cleveland Clinic, 9500 Euclid Ave, Cleveland, OH, 44195, USA

## Abstract

**Background:**

Chronic fatigue and inactivity are prevalent problems among individuals with multiple sclerosis (MS) and may independently or interactively have detrimental effects on quality of life and ability to participate in life roles. However, no studies to date have systematically evaluated the benefits of an intervention for both managing fatigue and promoting physical activity in individuals with MS. This study involves a randomized controlled trial to examine the effectiveness of a telehealth intervention that supports individuals with MS in managing fatigue and increasing physical activity levels.

**Methods/Design:**

A randomly-allocated, three-parallel group, time-series design with a social support program serving as the control group will be used to accomplish the purpose of the study. Our goal is to recruit 189 ambulatory individuals with MS who will be randomized into one of three telehealth interventions: (1) a contact-control social support intervention, (2) a physical activity-only intervention, and (3) a physical activity plus fatigue management intervention. All interventions will last 12 weeks and will be delivered entirely over the phone. Our hypothesis is that, in comparison to the contact-control condition, both the physical activity-only intervention and the physical activity plus fatigue management intervention will yield significant increases in physical activity levels as well as improve fatigue and health and function, with the physical activity plus fatigue management intervention yielding significantly larger improvements. To test this hypothesis, outcome measures will be administered at Weeks 1, 12, and 24. Primary outcomes will be the Fatigue Impact Scale, the Godin Leisure-Time Exercise Questionnaire (GLTEQ), and Actigraph accelerometers. Secondary outcomes will include the SF-12 Survey, Mental Health Inventory, Multiple Sclerosis Impact Scale, the Community Participation Indicator, and psychosocial constructs (e.g., self-efficacy).

**Discussion:**

The proposed study is novel, in that it represents a multi-disciplinary effort to merge two promising lines of research on MS: fatigue management and physical activity promotion. Collectively, the proposed study will be the largest randomized controlled trial to examine the effects of a lifestyle physical activity intervention in people with MS.

**Trial registration:**

NCT01572714

## Background

Multiple sclerosis (MS) is an immune-mediated neurological disease that results in a variety of consequences, most notably fatigue and physical inactivity. In fact, 75-90% of people with MS report having fatigue, and 60% describe it as their most disabling symptom [1-5]. Beyond fatigue, persons with MS are highly inactive compared with matched controls [6], and this inactivity may increase the risk of developing secondary conditions (e.g., obesity, heart disease, and diabetes) that can accelerate MS-related functional decline [7]. We further note that fatigue and physical inactivity may create a cyclical pattern of functional decline over time [8].

One approach for managing fatigue and physical inactivity involves the development of behavioral interventions that focus on teaching self-management tasks and skills [9]. For example, a randomized controlled trial (RCT) indicated that a fatigue self-management teleconference program was significantly more effective than a delayed-treatment control group for reducing fatigue impact and improving certain dimensions of health-related quality of life in persons with MS [10]. The changes were maintained for six months after the intervention. Another RCT demonstrated that a behavioral intervention delivered via the Internet was effective in promoting and sustaining physical activity behavior change in ambulatory adults with MS [11], and this effect was seemingly mediated by self-regulatory strategies of self-monitoring and goal-setting [12].

To date, interventions for managing fatigue and physical inactivity have typically been addressed as separate lines of research, even though these two common consequences of MS are likely interrelated [6,13,14]. The reciprocal relationship between fatigue and inactivity may accelerate a cycle of functional decline [15]. MS fatigue can decrease motivation to engage in physical activity [16,17]. Inactivity is associated with de-conditioning, mobility problems, depression, and further fatigue [18-21], which makes it even more difficult to engage in physical activity [22]. No study to date has systematically explored the combined benefits of teaching fatigue management strategies and promoting physical activity. We also note that the population with MS may have limited access to health professionals who can adequately address problems related to fatigue and inactivity, particularly for those who reside in rural communities [23,24]. Therefore, using a telehealth approach to address the reciprocal relationship between MS fatigue and inactivity could increase the intervention’s accessibility and dissemination.

The objective of this proposed study is to conduct a RCT to examine the effectiveness of a telehealth intervention that supports individuals in managing fatigue and increasing physical activity levels. The proposed study is novel in that it represents a multi-disciplinary effort to merge two promising lines of research on MS: fatigue management and physical activity promotion. The proposed fatigue management plus physical activity intervention (FM+) will consist of incorporating a teleconference fatigue management intervention informed by the work of Packer et al. [25], Finlayson et al. [10], and Mathiowetz et al. [26] with a novel, yet simple approach to promote lifestyle physical activity, i.e., encouraging goal-setting and self-monitoring with a pedometer. Our goal is to recruit 189 ambulatory individuals with MS who will be randomized into one of three telehealth interventions: (1) a contact-control social support intervention, (2) a physical activity-only intervention, and (3) a FM + intervention. Below we outline the aims, outcome measures, and associated hypotheses of the study.

### Specific aim 1

The first aim is to compare the effects of the three interventions on fatigue impact and physical activity levels. Outcomes will be the Fatigue Impact Scale (FIS) [27], the Godin Leisure-Time Exercise Questionnaire (GLTEQ) [28], and Actigraph accelerometers. Our hypothesis is that, in comparison to the contact-control condition, both interventions will yield significant improvements in FIS scores and physical activity levels, with the FM+ yielding significantly larger improvements.

### Specific aim 2

The second aim is to compare the effects of the three interventions on health-related quality of life, mental health, and participation in life roles. Outcomes will be the SF-12, Mental Health Inventory (MHI), Multiple Sclerosis Impact Scale (MSIS) [29,30], and the Community Participation Indicator (CPI) [31,32]. Our hypothesis is that, in comparison to the contact-control condition, both interventions will yield a significant increase in SF-12, MHI, MSIS, and CPI scores, with the FM + yielding a significantly larger increase.

### Specific aim 3

The third aim is to identify potential mediators of the interventions. Psychosocial constructs measures related to fatigue and physical activity (e.g., self-efficacy, goal-setting, and outcome expectations) will be administered to help identify the underlying mechanisms of the interventions’ possible effectiveness. Our hypothesis is that scores on these measures will significantly improve, and that improvements will explain the interventions’ effect on fatigue and physical activity.

## Methods/Design

A randomly-allocated, three-parallel group, time-series design with a social support program serving as the control group will be used to accomplish the aims of the study. Outcome measures will be administered at Weeks 1, 12, and 24. All interventions will involve a 12-week intervention period (three or six teleconference sessions weekly, and four one-to-one phone calls every other week) followed by a 12-week non-contact period to determine whether the effects persist. The design will enable us to compare the overall immediate and sustained outcomes of the three interventions. The protocol is approved by the Cleveland Clinic’s Institutional Review Board (ref: 11-844) in accordance with the Declaration of Helsinki. The study is funded by the National Multiple Sclerosis Society.

### Sample size

In order to achieve an adequate power of 0.80 to test hypotheses, we will seek to recruit a total of 189 individuals for this study and, more specifically, 63 in each condition. The total number of individuals to be recruited is based on calculations of sample size and power for a balanced repeated-measure mixed effect model with three time points (Weeks 1, 12, and 24) using a formula provided by Hedeker et al. [33]. We assumed equal variance and correlation (rho = 0.5) between measures across time points, and an effect size of 0.6 at Weeks 12 and 24. These calculations account for 20% attrition over time.

### Recruitment and eligibility

Individuals for this study will be recruited in several different ways in the State of Ohio. We have restricted recruitment to Ohio to avoid complications with state licensure laws. Fliers for the study will be distributed through MS support groups and National MS Society and MS Association of America events. We will recruit through physician referral and electronic health record review, and an MS patient registry maintained by the North American Research Consortium on MS (NARCOMS), which has approximately 400 registered individuals in Ohio who are ambulatory. Regardless of the recruitment method, individuals who are interested in participating in the study will be asked to contact the study office. Potential participants will undergo a telephone screening procedure to determine eligibility, and then a confirmation of eligibility through contacting their physician.

Inclusion criteria are: (1) physician-confirmed diagnosis of MS and physician-consent to initiate a physical activity program, (2) age between 18 and 65 years, (3) ability to walk at least 25 feet with a cane, (4) ability to carry on telephone conservations in English, (5) a Patient Determined Disease Step score between 1 (mild disability) and 5 (unilateral support required) [34], (6) a current sedentary lifestyle (i.e., purposeful exercises ≤ 2 days per week for 30 minutes) [35], and (7) experiences moderate to severe fatigue (a score of ≥ 4 on the Fatigue Severity Scale) [36]. Exclusion criteria are: (1) pregnancy, (2) cardiopulmonary diseases, (3) uncontrolled diabetes (hospitalized within the last six months), (4) greater than three falls in the past six months, and (5) severe cognitive deficits (weighted score of less than 12 on the short version of the Blessed Orientation Memory Concentration test) [37].

### Random allocation procedures

Randomization will be performed using a random permutated block design. The allocation will be performed using a random number generator. Three persons will be assigned to each block. The first person in the block will have an equal probability of being randomized into any of the three conditions. The second person will be randomized into one of the two remaining conditions and the third person in the block will automatically be placed into the third condition that has not been assigned.

### Intervention procedures

#### Physical activity-only intervention

This intervention will consist of three group teleconference sessions and four one-to-one calls. The overall purpose of the group teleconferencing will be to teach people how to engage in physical activity and set personalized goals to increase physical activity. The overall purpose of the one-to-one telephone calls will be to provide feedback and encouragement on achieving physical activity goals. Participants will first receive the three one-hour weekly group teleconference sessions from a licensed occupational therapist (OT). We selected OTs to deliver the teleconference sessions because of their expertise and training in managing group processes to facilitate behavior change. Participants will also be provided with four 10 to 20-minute, one-to-one, structured, follow-up telephone calls with a graduate assistant every other week. Both the group teleconference sessions and one-to-one follow-up telephone calls will be consistent with goal-setting theory [38] and increasing self-efficacy using verbal persuasion, teaching how to set measurable and reasonable goals, proving feedback on goals, and using role modeling (e.g., group members sharing stories) [39].

The first intervention session will focus on discussing the safety precautions for and benefits of physical activity specifically for persons with MS, and then teaching participants how to use a pedometer to monitor physical activity levels. Homework will be assigned to self-monitor walking behavior using the pedometer to generate a baseline level of physical activity for subsequent goal-setting. The second session will focus on using the pedometer to set goals. Participants will be encouraged to achieve personal goals related to the accumulation of pedometer steps by at least 1% every week. Participants will have the option to set additional goals related to exercise, which they can keep track of in their exercise log. Homework will be assigned to set personalized physical activity goals. The final session will focus on the personalized goals, and feedback from the OT and other group members on the appropriateness of goals. There will be a discussion and summary of the guiding principles that will help persons with MS safely engage in physical activity and overcoming barriers to engagement. The one-to-one follow-up telephone calls will focus on providing feedback on physical activity goals. Goals will be reviewed and adjusted biweekly under the guidance of a trained research assistant. The aim will be to teach the participant how to set and adjust goals independently.

#### FM+ intervention

Participants randomized into this intervention will first receive the three goal-setting physical activity teleconference sessions with the OT, and then will receive three additional teleconference sessions on fatigue management principles with the same OT. Thus, participants will receive a total of six group teleconference sessions. The participants will start to receive the four one-to-one calls from the research assistant after the completion of the first three session. For the purpose of this study, we will only teach fatigue management principles that can be integrated into the focus of changing physical activity behavior. This will include topics emphasizing the importance of rest throughout the day, setting priorities, activity analysis and modification, and living a balanced lifestyle. The first FM+ session will focus on teaching the guiding principles of fatigue management, and discussing the fatigue cycle and how inactivity and deconditioning can make fatigue worse. The second FM + section will focus on evaluating priorities, making active decisions, and using rest strategically in order to have adequate energy to engage in physical activity. The third FM + session will focus on teaching participants how to analyze and modify their schedules in order to manage their fatigue and achieve physical activity goals.

#### Contact-control social support intervention

The contact control group will consist of the same number of contacts as the FM+, i.e., six weekly one-hour teleconference calls with four biweekly one-to-one phone calls. Thus, this condition will allow us to determine whether it is the actual content being taught in the goal-setting physical activity intervention and FM+ intervention that is beneficial, rather than simply the contacts with the OT or social support provided by other group members. The teleconference calls and the one-to-one calls will involve a discussion of topics commonly addressed in support groups. Topics will include information on MS, disease modifying medications, preventive screening, community organizations, nutrition and supplements, cognitive problems, and recognizing symptoms of depression and chronic stress. For the teleconference sessions, participants will be given the opportunity to share problems and provide peer support and solutions for others. For the one-to-one calls, participants will be asked about topics of interest, and the graduate student will gather educational materials and send them to the participant. This information will be the basis for the discussion in the calls. When issues related to fatigue and physical activity are raised, participants will be directed to publically available educational pamphlets. This type of social support group has been used successfully in other clinical trials as a control condition to evaluate the efficacy of fatigue management programs in persons with MS [40].

##### Ensuring the fidelity of the interventions

Guidelines suggested by Bellg [41] will be implemented to promote uniform application of all three interventions. For example, a manual of operating procedures will be provided to interventionists and incorporated into training. Interventionists’ adherence to the intervention protocol will be verified using a checklist and episodic monitoring of the sessions over the phone. Fidelity of the intervention “dose” will be further monitored by tracking attendance for each session, monitoring the completion of worksheets, and testing comprehension.

##### Primary dependent variables

All dependent variables will be administered at baseline, at the end of the 12-week intervention period, and at a 24-week follow-up period through the mail.

Fatigue impact will be measured using the Fatigue Impact Scale [27]. This 40-item scale evaluates the perceived impact of fatigue on everyday life, and is valid and reliable among people with MS. It was developed by interviewing patients with MS, and has discriminant validity between patients with MS, general fatigue, and essential hypertension [27]. Respondents rate each statement using a five-point Likert-type scale ranging from 0 (no problem) to 4 (extreme problem). A total score and three subscale scores (physical, social, cognitive) can be produced from participants responses. The FIS was used to detect changes in fatigue impact resulting from a fatigue management teleconference program [10].

Physical activity will be evaluated using the Godin Leisure-Time Exercise Questionnaire (GLTEQ) [28] and an Actigraph tri-axis accelerometer. The GLTEQ is a self-administered measure of usual physical activity. The GLTEQ includes three items that measure the frequency of strenuous, moderate, and mild exercise for periods of more than 15 minutes during one’s free time in a typical week. Participants will be sent an accelerometer to wear daily over three seven-day periods (baseline, post-test, and follow-up). Data are retrieved for analysis via a computer and software provided with the unit. We will use an epoch of one minute. Participants will be instructed to wear the accelerometers all day, except while showering and swimming, for each of the three seven-day measurement periods. The participant will use a log to record the time that the accelerometer is worn, and this will be verified by inspection of the minute-by-minute accelerometer data. Motl et al. have demonstrated the validity and reliability of both the GLTEQ and the accelerometer in the population with MS [42].

##### Secondary dependent variables

Health-related quality of life, mental health, and participation in life roles will be measured with the SF-12, Mental Health Inventory, and Community Participation Indicator (CPI), respectively. The SF-12 and Mental Health Inventory are valid and reliable measures, and are commonly used in the population with MS [43]. The CPI is a new measure developed by Heinemann and colleagues. The measure was developed with input from multiple stakeholders, and has been validated through Rasch analysis in a sample of 1,163 individuals with a variety of disabling conditions [31,32]. In a subsequent online survey study, the measure had good test-retest reliability over a 2.5 month period in people with MS (t = 0.12, p = 0.91; r = 0.84, p < 0.01). For each item, respondents rate the frequency of engagement (either in days, hours, or times per week, depending on the type of activity), whether it was important (yes/no), and whether they were doing it not enough, enough, or too much. Examples of the 20 items included are: work for money, provide care for a loved one, volunteer, and participate in learning activities.

Six questionnaires will be implemented to identify underlying mechanisms of the interventions’ possible effectiveness for increasing physical activity levels and reducing fatigue impact. Psychosocial construct measures for physical activity will be the Self-Efficacy for Exercise Scale [44], Exercise Goal-Setting Scale [12,45], and Multidimensional Outcome Expectations Scale [46]. All three questionnaires are validated and have been used in previous MS research on physical activity [45,47]. Psychosocial construct measures for fatigue management will be the validated Self-Efficacy for Performing Energy Conservation Strategies [48] along with two additional measures on goal-setting and outcome expectations, which will be developed and validated during the study.

To address potential confounders on the interventions’ effects, information will be collected from participants for the purposes of post-hoc sub-group comparisons and potential use as control variables. These variables will include age, sex, educational level, medications, exacerbations, functional level, and involvement in any other wellness, rehabilitation, or exercise program. Information about changes in MS-related disability, exacerbations, medications, use of rehabilitation programs and other types of exercise/educational programs, and reasons for attrition will be gathered over the phone at 12 weeks and at the 24-week follow-up.

##### Data management and analysis

A secure online application, Research Electronic Data Capture [49], will be used to manage recruitment efforts and facilitate data collection. The first step in the analysis process will be to compute summary statistics, calculate outcome scores, and conduct quality control assessments. The second step in the analysis will be to test whether the randomization process resulted in equivalent groups at baseline. If differences are found at baseline, these variables will be used as covariates during the hypothesis testing phase. The third step in the analysis process will be addressing normality assumptions, and the potential for missing data, before hypothesis testing begins. If the normality assumption is violated, then an appropriate data transformation will be used. Missing data will be treated conservatively. If appropriate, maximum likelihood, multiple imputation, or last observation carried forward will be used to estimate the missing values. After addressing issues of attrition and missing data, the fourth step will be to conduct intent-to-treat analysis to test the hypotheses. Linear mixed-effects models will be used as the primary analysis tool for testing hypotheses, which include condition, time, and interactions as the fixed covariates, and a random intercept that models the correlation among outcomes collected from the same subject. The mixed models will allow us to determine whether there are significant differences between the three conditions and whether there are significant differences across time. All tests will be two-sided, and p = 0.05 will serve as the criterion for significance. As is typically the case for mixed-effects analyses, the results will not be controlled for multiple comparisons. The generalized estimating equation approaches will be used if the normality distributional assumption is violated for the random effects in the mixed models. To test for mediation, we will use a linear regression model. This analysis will first involve regressing changes in fatigue impact or physical activity on the intervention conditions, and then regressing changes in fatigue or physical activity on the intervention conditions plus changes in the potential mediator. Mediation will be based on the interventions’ association with physical activity or fatigue impact being reduced in magnitude and non-significant after controlling for the mediator variable.

## Discussion

Possibly the most overlooked determinant of a poor quality of life and participation restrictions in adults with MS involves the cyclical relationship between fatigue and inactivity, and the subsequent development of secondary conditions. Research indicates that fatigue is a common and profound barrier to engaging in physical activity among persons with MS [16,17], and that inactivity is related to functional decline [50]. Furthermore, in a recent longitudinal study, Motl et al. [18] demonstrated that changes in both fatigue and depressive symptoms are significantly associated with reduced physical activity levels. Finally, we note that many studies indicate that MS fatigue and inactivity negatively affect quality of life and participation in life roles [51-55]. Together, these studies suggest a cyclical relationship between fatigue and inactivity, and that it reduces quality of life and increases participation restrictions. Thus, there is a need to conduct cross-disciplinary research that fills the existing gap in the MS literature regarding whether combining fatigue management with physical activity promotion has beneficial synergistic effects on increasing quality of life and reducing participation restrictions in persons with MS.

The proposed interventions are expected to have beneficial effects on participants regardless of the cause of MS fatigue. Although the pathophysiology of MS fatigue remains unclear, researchers distinguish between primary and secondary causes of MS fatigue [14]. Primary causes of fatigue pertain to MS pathology and adaptive responses, such as brain lesions, axonal damage, cortical reorganization, and neuroendocrine factors [56-58]. Secondary factors in fatigue pertain to personal characteristics that are not specific to MS, such as de-conditioning and depression. Ultimately, participants in the proposed study may experience fatigue because of primary and secondary factors. The fatigue management program involves teaching compensatory strategies, and physical activity may mitigate secondary factors associated with fatigue [47]. Thus, we expect the FM+ intervention to be the most effective because of its potential to facilitate engagement in compensatory strategies and address secondary fatigue factors.

Although physical activity may prevent de-conditioning and improve other secondary factors associated with fatigue, such as depression, there is limited research on identifying cost-effective strategies to increase physical activity levels. Encouraging participants over the phone to set realistic goals and self-monitor walking behavior with a pedometer may be a cost-effective and ecologically-valid strategy for increasing physical activity behavior in the population with MS. Since the most common type or mode of physical activity among persons with MS is walking [6,59], having participants set goals to increase daily walking steps is a practical method for increasing physical activity. Furthermore, Stuifbergen et al. [60,61] have demonstrated the utility of using goal-setting to promote healthy behaviors in persons with MS. Locke and Latham contend that goal-setting directs attention toward goal-relevant activities and away from irrelevant activities, and helps foster intrinsic motivation [38]. They posit that personal barriers can impact the efficacy of goal-setting for motivating behavior change. Thus, the development of strategies for overcoming such barriers as fatigue might augment the effect of a goal-setting approach for increasing physical activity.

Nonetheless, we acknowledge there is the risk that a walking program may be ineffective in adults with MS, or that a walking program may result in imprudent risk for injuries. Cold and icy or hot weather, along with other environmental factors such as connectivity of sidewalks, could influence individuals’ ability and willingness to walk. This potential limitation will be addressed by providing information on how individuals with MS can safely engage in a walking program on a regular basis. For example, tips will include taking a cool shower before engaging in physical activity, wearing a cooling vest while engaging in physical activity, or walking in a climate-controlled facility, such as in a mall. We will monitor the frequency of injuries and exacerbations, as well as overall symptom severity and function, with a safety questionnaire. However, moderate-intense physical activity programs are generally considered to be safe for individuals with mild to moderate MS symptoms [62].

## Conclusions

This study will build upon the work of Stuifbergen et al. [60], McAuley et al. [35], and Motl et al. [45] by implementing a minimal-contact telephone-based intervention; the work of Mathiowetz et al. [26] and Finlayson et al. [63] by evaluating the combined benefits of promoting physical activity and teaching fatigue management strategies; and the work of Bombardier et al. [64] by exploring the effectiveness of changing behavior using a combination of group teleconferences and one-to-one calls without any face-to-face contact. This proposed study represents the largest randomized controlled trial to examine the effects of a lifestyle physical activity intervention in people with MS, and the first study to systematically explore the combined benefits of teaching fatigue management strategies and promoting physical activity. If the teleconference intervention is shown to be effective, future translational research will involve exploring strategies to implement it within clinical care and comparing it to traditional in-person, individualized rehabilitation interventions.

## Abbreviations

MS: Multiple sclerosis; RCT: Randomized controlled trial; FM +: Fatigue management plus physical activity intervention; MHI: Mental Health Inventory; MSIS: Multiple Sclerosis Impact Scale; CPI: Community Participation Indicator; NARCOMS: North American Research Consortium on MS; OT: Occupational therapy.

## Competing interests

The authors declare that they have no competing interests.

## Authors’ contributions

MP contributed to the conception and design of the study and drafting the manuscript. He will also oversee data acquisition, analysis, and interpretation of data. MF contributed to the conception and design of the study and drafting the manuscript. She will also oversee the training of occupational therapist and contribute to data analysis and interpretation. RM contributed to the conception and design of the study and drafting the manuscript. He will provide expertise in analyzing accelerometer counts, psychosocial construct measures, and mediating/moderating effects. FB contributed to the revisions of the manuscript for important intellectual content. He will provide expertise in analyzing health and function measures and serve as a medical consultant. All authors read and approved the final manuscript.

## Authors’ information

MP’s training is in rehabilitation and behavior science. He is currently a project scientist at the Cleveland Clinic in the Departments of physical medicine and rehabilitation and biomedical engineering. MF is a licensed occupational therapist as well as professor and director at Queen’s University in School of Rehabilitation Therapy. RM is an associate professor in the Department of Kinesiology and Community Health at University of Illinois Urbana-Champaign. FB is a physiatrist and director of rehabilitation services at the Cleveland Clinic Mellen Center for Multiple Sclerosis Treatment and Research.

## Pre-publication history

The pre-publication history for this paper can be accessed here:

http://www.biomedcentral.com/1471-2377/12/122/prepub
